# Assessing the Merits and Effectiveness of Peer Teaching in Small Groups through the Employment of Different Learning Media

**DOI:** 10.1055/s-0043-1776044

**Published:** 2023-10-30

**Authors:** Saurabh RamBihariLal Shrivastava, Prateek Saurabh Shrivastava

**Affiliations:** 1 Department of Community Medicine, Datta Meghe Medical College, Off-campus Centre of Datta Meghe Institute of Higher Education and Research, Nagpur, Maharashtra, India

**Keywords:** peer teaching, learning media, student-centered, self-directed learning

## Abstract

**Background** Peer teaching is a well-acknowledged method to facilitate teaching and learning among medical students. The objective of the study was to assess the merits and effectiveness of peer teaching in small groups using a student-centered approach through the employment of different learning media.

**Methods** This was a cross-sectional descriptive study conducted among a group of 34 students from third professional year. Purposive sampling was used, wherein students were subdivided into five small groups. At the beginning, a pretest consisting of 10 multiple-choice questions was conducted on the topic “Epidemiology of Hypertension.” This topic was further subdivided into five subtopics, and five separate learning media (viz., video, PowerPoint, white board, chalk-and-talk, and chart) were allocated using the lottery method. Each group discussed the allotted topic and then presented their findings in the large group using the assigned learning media, while other groups gave feedback, and the best group was decided through multivoting. Posttest was administered and the results were compared with the pretest. Data entry was done in Microsoft Excel and analysis was done using SPSS 16. Descriptive statistics and paired *t* -test was used to compare the results in pretest and posttest at *p* level < 0.05.

**Results** This innovative session of peer teaching featured 34 students, with a mean age of 22 ± 1.3 years. While carrying out the Kirkpatrick Level 1 evaluation, almost 90% students liked the role of the facilitator in stimulating interest in the topic. There was a mean average increase of 1.7 marks on comparing the scores of posttest with pretest (Kirkpatrick Level 2), and the reported difference was statistically significant. Joyful learning 24 (66.7%) and group discussion 23 (63.9%) emerged as the most liked aspects of the session.

**Conclusion** In conclusion, peer teaching through learning media is an effective method as it promotes active learning, improves communication skills, and improves the academic performance of students. Peer teaching using different learning media proved to be quite effective in improvement of knowledge about epidemiology of hypertension, the pros and cons of different learning media, and encouraged creativity among students.

## Introduction

Peer teaching is a well-acknowledged method to facilitate teaching and learning among medical students as it provides them with a platform to interact with each other and learn in a collaborative manner. 1 Peer teaching gives medical students a chance to teach their peers (student-centered), and in the process learn the art to simplify complex concepts and even deliver constructive feedback to them about their learning. 1 2 At the same time, it provides students with a supportive and enabling learning environment, wherein students participate in the learning process actively and thus accounts for better retention of knowledge and attainment of intended learning outcomes. 2 3 Further, peer teaching has the potential to bring about an overall improvement in the quality of medical education delivered to students as it augments the learning experiences among medical students. 1 2 3 The learning pyramid suggests that once we teach others (peer teaching), the average retention rate is 90% which is much more than any other form of learning. 4 Moreover, peer teaching also supports the learning theories of behaviorism (wherein depending on the peer teaching, other students acquire knowledge or skills—change in behavior), constructivism (peers build upon the knowledge that students already have), etc.

Peer teaching has been employed in heterogeneous settings (such as small group discussions, clinical teaching, problem-based learning, etc.) by different researchers and the studies have given encouraging results in terms of better acquisition of knowledge and performance in assessments. 1 3 5 6 7 In peer teaching, the general fear associated with committing a mistake in a teacher-student setting is significantly reduced. 4 5 In other words, they feel comfortable and safe to learn from their peers without being afraid of worrying about the consequences, if they commit a mistake. 4 5 Another advantage of peer teaching is that it can neutralize the challenge of a shortage of learning resources (viz., through encouraging students to make use of existing resources like sharing of textbooks, notes, and online collaborative tools; knowledge sharing and broader dissemination of information; creation of supplementary materials by the peer teachers that can enhance the learning experience for everyone; etc.), and at the same time give them an opportunity to enhance their communication skills. 3 4 5 This entire process is not only useful for the students but even for the teachers as they gain insights on different ways a topic can be taught, especially keeping in mind how the students of the current generation learn. 6 7

Considering the fact that medical education is complex, wherein students are expected to learn multiple competencies, learning media occupy a crucial place as they aid the students to acquire the intended learning competencies expected of a medical graduate. 8 The need of the hour is to employ a combination of learning media that will neutralize the monotony linked with conventional media and will give students a chance to actively engage, promote better retention, and stay motivated in the learning process. 8 Further, learning media also has the potential to bridge the gap that exists between theoretical concepts and practical relevance and thus provide better understanding to the students.

This calls for the need that we must employ appropriate and combination of learning media (viz., videos, simulation, chart, PowerPoint, etc.), that not only meets the need of students with different learning styles, but act as an effective tool to acquire critical thinking, problem-solving, and practical skills. 8 9 Thus, keeping the wide range of merits that have been attributed to peer teaching that enables student-centered learning and the scope of learning media in enhancing the learning experiences among medical students, the current study had been planned with objectives to assess the merits and effectiveness of peer teaching in small groups through the employment of different learning media.

## Materials and Methods

*Study design and study settings* : This was a cross-sectional descriptive study conducted among a group of 34 students from the third professional year in the department of community medicine in a tertiary medical college as a part of a small group teaching session. The selected topic for peer teaching was “Epidemiology of Hypertension.” These students are a part of the undergraduate medical training course which is of 5.5 years' duration, including 1 year of internship. The 4.5 years of the course is divided into first, second, third professional year—part I, and third professional year—part II, and each year had specific number of subjects that a student must clear in summative assessment. Community medicine is a subject that is taught to students right from the first professional year, but its summative assessment finally happens at the end of the third professional year—part I.

*Sampling method* : Purposive sampling was employed in the study, wherein we selectively those third professional year medical students who were posted in the department during the period of study.

*Inclusion and exclusion criteria* : All students posted in the department for the small group session were included. Only those students who were absent during the session were excluded from the study.

*Data collection* : The entire session was facilitated by a single faculty to provide guidance to the students whenever they are in doubt and also to streamline the entire peer teaching session. At the beginning of the session, a pretest consisting of 10 multiple-choice questions (MCQs), each carrying 1 mark, was conducted on “Epidemiology of Hypertension” using Google Form. The MCQs were of the single best type with four options and tested recall and understanding levels as per Bloom's taxonomy of the cognitive domain. All these questions were designed after a thorough brainstorming session in the department, and subsequently it was pilot-tested among a representative group of students. Further, based on the feedback obtained, the questions were modified and this validated set of MCQs were used for both pretest and posttest. Subsequently, the facilitator explained the objectives of the small group teaching session and all 34 students were divided randomly into 5 groups by asking them to call out from 1 to 5, and subsequently, all similar numbers were grouped together (each group comprising of 6–7 members each). Further, the lottery method was used to allocate the topic as well as the learning media to each of the groups by inviting one representative from each of the groups (as mentioned in Table 1 ).

**Table 1:** Group-wise allocation of topic and learning media

Group	Allocated topic	Allocated learning media
1	Distribution and magnitude of hypertension, and tracking of blood pressure	Video
2	Risk factors and rule of halves	PowerPoint presentation
3	Prevention of hypertension	White board
4	National Programme for Prevention and Control of Cardiovascular Disease, Cancer, Diabetes, and Stroke	Chalk-and-talk
5	Five levels of prevention	Chart

Each group was provided with a time of 45 minutes to discuss the topic and then present their discussion findings in the large group using the allocated learning media. Moreover, students were given instructions to sit in the form of a circle and discuss their plan of action and execution. They were also asked to identify leaders and presenters from the group and even define the role of each member in the group. Students were given the freedom to refer to any learning resource (viz., textbook, government documents, World Health Organization web site, etc.) for the allotted subtopic and at the end of the discussion, make a presentation to the entire class within 5 minutes.

At the time of the presentation by one group, other groups were asked to ask questions/clarifications and also provide constructive feedback on what was done well and the areas wherein the group could have done better. Subsequently, the best group was decided based on multivoting, wherein each student was given an opportunity to vote three times in favor of the group which they liked the most (except their own group). This voting was done keeping in mind variables like teamwork (representation from each member of the group), presentation skills (such as clarity of presentation, logical flow, etc.), overall outcome (like quality of video, clarity of chart/whiteboard/blackboard, adherence to standard rules in making PowerPoint, etc.), and the ability to respond to the questions of peers from other groups.

Upon the completion of the session, a posttest was administered to the students using the same 10 MCQs and the results were compared with the pretest. At the end of the session, students were asked to provide feedback about the peer teaching session and using a Google feedback form, which was designed to obtain sociodemographic details, feedback on attributes of the session on a Likert scale of 1 to 5 (strongly disagree to strongly agree), feedback on the facilitator, and two open-ended questions (viz., mention any three things that you liked in today's peer teaching session, and mention areas that needs to be improved in the future).

The Kirkpatrick Level 1 (reaction) was assessed by obtaining the immediate reactions of the students to the session like what they liked, what needs to be improved, and feedback of session attributes. This eventually provided insights about the utility/merits of the peer teaching session. For the Kirkpatrick Level 2 (learning), we carried out a comparison between the score of pretest and posttest, and this gave insights about the extent of learning after the session. In other words, this comparison provided inference about the effectiveness of the peer teaching session.

*Study variables* : Feedback from students about the role of the facilitator in stimulating interest in the topic, provision of adequate opportunity to participate in group activity and discussion, topic coverage, time utilization, adherence to session objectives, merits of peer teaching, areas that need to be improved, etc.

*Statistical analysis* : Data entry was done in Microsoft Excel sheet and analysis was done using SPSS version 16. Descriptive statistics were used to represent the data in the form of mean, standard deviation, frequency, and percentages. Paired *t* -test was used to compare the performance of the students in pretest and posttest at *p* level < 0.05. Open-ended answers were grouped into categories (similar kinds of responses are grouped together and presented in the form of cumulative numbers and percentages).

*Ethical considerations* : Written informed consent was obtained from the study participants before the conduct of the study. Students were given reassurance that performance in the assigned task will not have any impact on their academic performance and the entire exercise is being done to encourage learning and as a part of formative assessment.

## Results

**Fig. 1:**
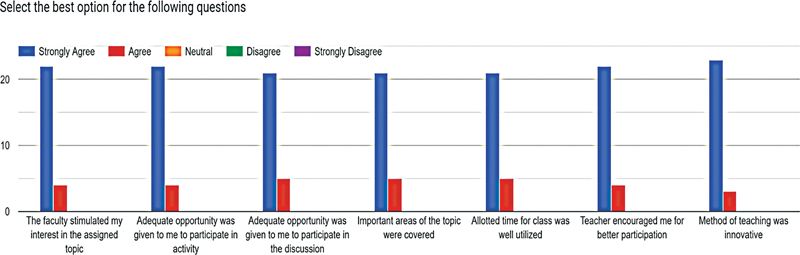
Feedback on attributes of the session.

This innovative session of peer teaching on the different subtopics related to epidemiology of hypertension were undertaken as a part of a 3-hour small group discussion session. A total of 34 students, including 21 (61.8%) girls and 13 (38.2%) boys, with a mean age of 22 ± 1.3 years participated in the peer teaching session using five different learning media. While carrying out the Kirkpatrick Level 1 evaluation of the session using a 5-point Likert scale, it was quite encouraging to note that almost 90% of the respondents positively rated the domains of the role of the facilitator in stimulating interest in the topic, provision of adequate opportunity to participate in a group activity, and overall encouraging experience in the teaching-learning process ( Fig. 1 ). There was a mean average increase of 1.7 marks on comparing the scores of the posttest with the pretest and the reported difference was statistically significant (Kirkpatrick Level 2).

**Fig. 2:**
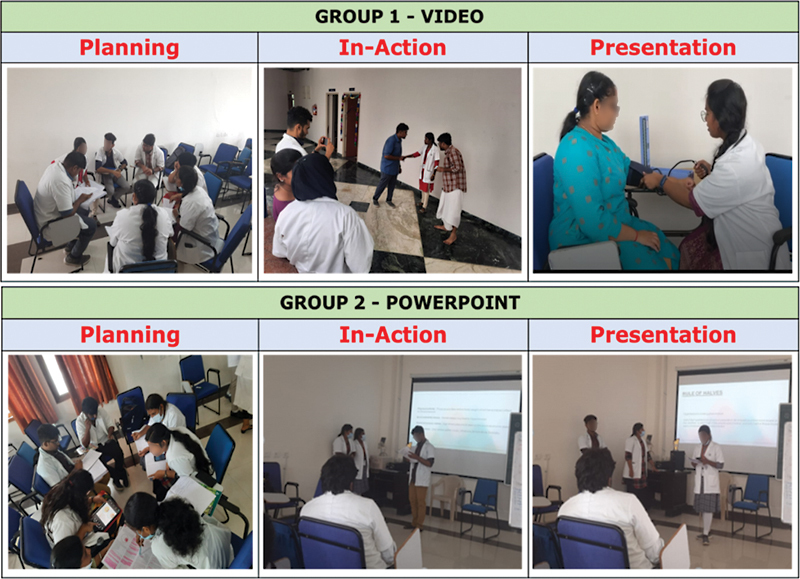
Activity by group 1 and group 2.

Group 1 (7 members) was assigned to the task of preparing a video to explain the “distribution and magnitude of hypertension and tracking of blood pressure” ( Fig. 2 ). The group came out with a 90-second video, wherein all members were assigned different roles and the video was appreciated by everyone owing to the clear message that was delivered and that it was made in a very short period of time. Group 2 consisting of 7 members was asked to make a PowerPoint presentation on the topic “Risk Factors and Rule of Halves,” and then make the presentation to the large group ( Fig. 2 ). Based on the constructive feedback that was given to the group, it was highlighted that all team members could have been involved in the presentation, as only three of them made the presentation.

**Fig. 3:**
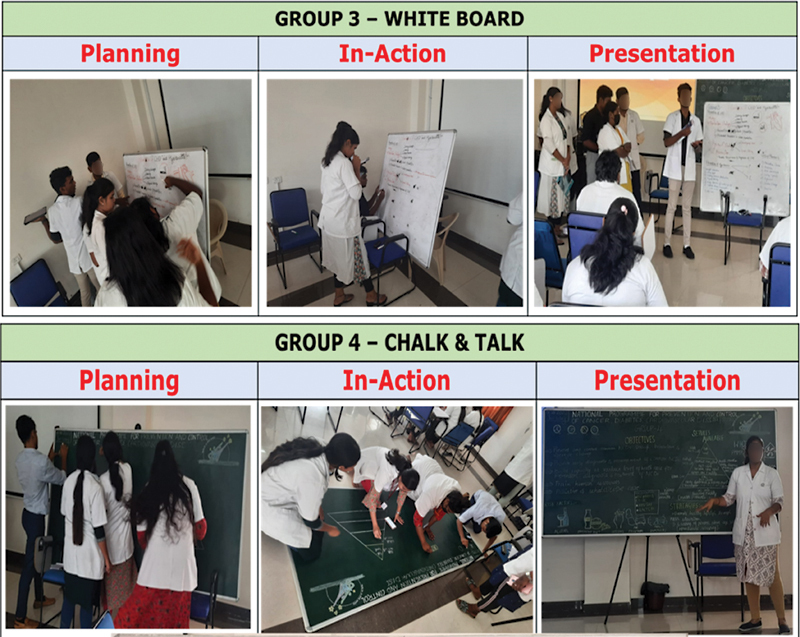
Activity by group 3 and group 4.

Group 3 comprising of 6 students was given the task to present about the “prevention of hypertension” to the large group using a white board ( Fig. 3 ). The group received positive feedback from other groups, but the members were not able to answer about quaternary prevention and its need in public health. A total of 7 students was allocated to group 4, which was assigned the task to elaborate on the “National Programme for Prevention and Control of Cardiovascular Disease, Cancer, Diabetes, and Stroke” using chalk-and-talk as the learning media ( Fig. 3 ). The overall content prepared by the group members was appreciated by all other groups, nevertheless as a constructive suggestion, it was emphasized that all members should be engaged in the presentation (as only one student presented the entire topic).

**Fig. 4:**
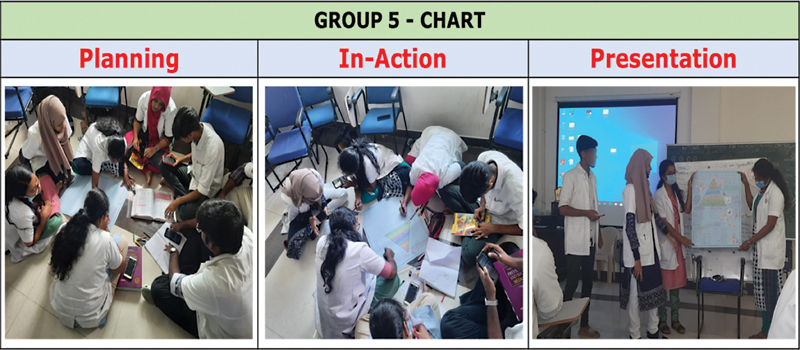
Activity by group 5.

**Fig. 5:**
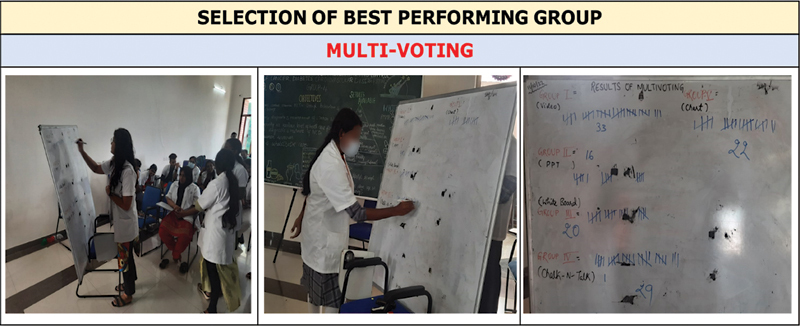
Multivoting to decide the best group.

Group 5 was allocated the task to present “Five Levels of Prevention” using a chart as the learning media ( Fig. 4 ). The overall outcome was colorful and attractive, but some of the members from other groups mentioned that the details in the chart were not visible. For the benefit of the undergraduate students, after the presentation of each group, the pros and cons of different learning media were discussed by the facilitator to help them make an informed choice in their future assignments. Finally, to decide the winner among all the groups, multivoting was done ( Fig. 5 ).

Table 2 depicts the areas which the students liked about peer teaching and the most frequently cited options were joyful learning 24 (66.7%), peer teaching 23 (63.9%), group discussion 23 (63.9%), and do's and don'ts about learning media 22 (61.1%), respectively. With reference to constructive suggestions for improvement in the peer teaching session, 7 (19.4%) students of the sessions suggested more time for preparation while 11 (30.5%) students asked for similar sessions in the future to augment their learning.

**Table 2:** Feedback on positive things in the peer teaching session

Parameters	Number a (%)
Interactive session	19 (52.8)
Equal opportunity was given to all students	11 (30.5)
Understanding and learning about the topic was made easier	16 (44.4)
Group discussion	23 (63.9)
Involvement of all students	18 (50)
Feedback from other teams	15 (41.7)
Informative and resourceful	19 (52.8)
Joyful and fun-filled learning	24 (66.7)
Innovative approach to teach a dry topic	17 (47.2)
Teamwork and cooperation	17 (47.2)
Time management	9 (25)
Encouragement of creativity	7 (19.4)
Interesting to learn from batch mates (peer-teaching)	23 (63.9)
Improvement in communication	11 (30.5)
Do's and Don'ts about learning media	22 (61.1)
In-depth coverage of topics	19 (52.8)

a Responses obtained were not mutually exclusive.

## Discussion

The peer teaching session was conducted for third professional year students to cover a topic on the epidemiology of hypertension using five different learning media. On a similar note, the findings of a study done with an intention to identify the preference of medical students for peer teaching, 62.5% of the third professional year students opted for small group teaching. 10 The current study was first of its kind as it involved the employment of five different readily available learning media to promote collaboration among team members and also to ensure an in-depth understanding of the topic. A total of 34 students, including 21 girls and 13 boys actively participated in the session to represent subtopics allocated to their groups. The findings of a study done on peer teaching involving 33 students in the United States revealed that students got multiple opportunities to enhance their professional growth. 5

In our study, 24 (66.7%) participants mentioned that the peer teaching session was joyful and fun-filled. This could be because we conducted the session in the form of a competition, wherein it was announced that the best performing group will be felicitated. This accounted for a sense of competition among groups and all of them ensured that their group presentation remains innovative and different from the usual. However, the findings from a study done among first year medical students from Turkey revealed that the major benefit of peer teaching was in terms of improvement in the confidence level of students. 4 This could be due to the fact that all first year students are very much new to the stream of medicine and they do not have much experience in talking about medical subjects, in contrast to our study, wherein participating students were from the third professional year who already had in past experience for public speaking.

The present study clearly highlighted the utility of peer teaching as 23 (63.9%) students reported that it was interesting to learn from their peers and the same number of students also attributed their learning to the group discussion that happened in the preliminary stage of the assignment. On a similar note, multiple other studies have reported that peer teaching makes the entire learning more fruitful with better acquisition of knowledge and improved retention among them. 1 2 3 4 5 6 This could be due to the active engagement of the students in the entire exercise, which envisages a student-centered learning approach. Moreover, we cannot also rule out the role of self-directed learning that happens between students once they were given a topic and this would have surely helped them in their learning process.

The findings of a longitudinal study done at the University of Belgium reported that students who participated in peer teaching activities had better academic performance at different points of time during their undergraduate training when compared with other students from the same cohort. 6 Similar kind of results were obtained in another study done at a medical university in Germany that involved medical students from the first professional year. 11 Even in our study, there was an average increase of 1.7 marks and the obtained results were found to be statistically significant. Moreover, we must note that in the study done in Wardha in the department of physiology and even in the present study, the importance of feedback was emphasized to augment learning as it aids the students to understand their strengths and the areas where they need to improve upon. 7

The findings of a study done in Ireland among first year anesthesia residents revealed that the majority of the students opined that peer teaching plays a defining role in improving communication skills. 12 Similarly, in our study, 11 (30.5%) students reported that peer teaching as a teaching method is effective in augmenting communication skills. The impact on communication skills could be due to the interactions which the students had in small groups while they were preparing for the assignment, the interactions they had while they made presentations to the large group, and the interactions reported while they gave feedback to all other groups after their presentation. In other words, peer teaching followed by feedback administration gave them multiple opportunities to improve their communication skills.

The findings of a study done with the intention to explore the acceptability of peer teaching as a teaching method at the University of New South Wales revealed that the method was very much acceptable by the students. 13 At the same time, students found the method to be very effective and an approach that could be implemented even in other settings. 13 Along similar lines, in the present study, it was reported that the peer teaching session was interactive (19; 52.8%), gave equal opportunity to all students (11; 30.5%), and that understanding and learning about the topic was made easier (16; 44.4%). All these results indicate that during these sessions, the student gets multiple opportunities to learn and retain the knowledge and become a better version of themselves. 13 14 15

In the current study, medical students used only five learning media (viz., video, PowerPoint presentation, white board, chalk-and-talk, and chart) to present the assigned subtopic. However, in a study done in a medical college in Germany, among students while reading pharmacology, a variety of learning media were used. 16 Similarly, the augmented reality tool was employed by students to enhance their physical examination skills of pregnant women in Indonesia. 17 This could be primarily because of the fact that in our study, we restricted the number of learning media to only five to make the entire process feasible and practical, while that was not the case in other studies. 16 17

The strength of the present study was that to ensure that small group discussion sessions are more effective, we grouped students into further smaller groups to ensure better collaboration between team members. Subsequently, in each group, we instructed members to identify a leader and specify roles for each member and this was done to ensure that all members are aware of their responsibilities and at the same time accountable. Further, it was informed to all students that whenever one group is presenting, all the other groups must ask questions and suggest areas where the group did well and the areas where they could have done better. This was done with an intention to promote collaborative learning and to ensure that students receive feedback from their peers (more readily accepted than by teachers). The study had its share of limitations, as it was done in only a single batch of students, that too, only for a single topic, and thus study results cannot be generalized.

## Conclusion

In conclusion, peer teaching is an important and valuable method of teaching and learning in medical education, as it promotes active learning, ensures an in-depth understanding of the topic, improves communication skills, and even brings about improvement in the academic performance of the students. Based on the findings of the current study, it was concluded that peer teaching using different learning media proved to be quite effective in the improvement of knowledge about epidemiology of hypertension, the pros and cons of different learning media, and encouraged creativity among students.
